# Combined effects of mechanical overload and high‐intensity interval training on skeletal muscle hypertrophy in male mice

**DOI:** 10.14814/phy2.70542

**Published:** 2025-09-12

**Authors:** Hayato Shinkai, Takanaga Shirai, Kazuki Uemichi, Ryoto Iwai, Tomohiro Iwata, Riku Tanimura, Shunsuke Sugiyama, Tohru Takemasa

**Affiliations:** ^1^ Graduate School of Comprehensive Human Sciences University of Tsukuba Tsukuba Ibaraki Japan; ^2^ Department of Human Sciences Kanagawa University Yokohama‐shi Kanagawa Japan; ^3^ Research Fellow of Japan Society for Promotion Science Chiyoda‐ku Tokyo Japan; ^4^ Research Organization of Science and Technology Ritsumeikan University Kusatsu Shiga Japan; ^5^ Faculty of Health and Medical Sciences Kyoto University of Advanced Science Kameoka‐shi Kyoto Japan; ^6^ Institute of Health and Sport Sciences University of Tsukuba, University of Tsukuba Tsukuba Ibaraki Japan

**Keywords:** high‐intensity interval training, hypertrophy, mTOR signaling, skeletal muscle

## Abstract

Athletes often perform concurrent training that combines different exercise modalities. It is believed that muscle hypertrophic adaptation is inhibited by endurance exercise; however, the molecular mechanisms underlying the effects of high‐intensity, short‐duration exercise on muscle hypertrophic responses remain unclear. In this study, we determined the effects of high‐intensity interval training (HIIT) on mechanical overload‐induced muscle hypertrophy in mice. Eight‐week‐old male mice were divided into the following three groups (*n* = 6–8): Sham surgery (Sham), myotenectomy‐induced mechanical overload (OL), and OL with HIIT by forced swimming (OL + HIIT). After 4 weeks of intervention, the OL + HIIT group exhibited an increase in plantaris muscle weight and muscle fiber cross‐sectional area as well as the OL group. The OL + HIIT group showed a similar increase in mTOR downstream proteins rpS6, S6K1, and 4E‐BP1 phosphorylation compared with the OL group. AMPK was activated by HIIE and may play an inhibitory role by attenuating mTOR. To clarify its involvement, acute phase experiments were conducted to evaluate mTOR signaling immediately after a single round of HIIE. The increase of rpS6 and S6K1 phosphorylation was unchanged after a single HIIE exposure, despite AMPK upregulation. Our results suggest that interference effects induced by HIIE may not occur in mechanically overloaded mouse skeletal muscle.

## 1 INTRODUCTION

Skeletal muscle is a plastic tissue, and specific adaptations are induced depending on training modality (Atherton et al., 2005). To improve athletic performance, athletes must meet different physiological demands, such as muscle hypertrophy and oxidative and glycolytic metabolic capacity. Therefore, many athletes perform training combined with different modalities, such as resistance and endurance training, which combines different exercise modalities and is referred to as concurrent training (CT) (Hickson, 1980; Ogasawara et al., 2014). Endurance exercise can attenuate the hypertrophic adaptations induced by resistance training, a phenomenon referred to as the interference effect (Wilson et al., 2012). Although the extent and specific conditions under which this effect occurs in humans remain unclear, rodent models have demonstrated this interaction more consistently, providing a useful framework for investigating the underlying molecular mechanisms (Ogasawara et al., 2014; Shirai et al., 2020; Shirai, Hanakita, et al., 2021; Shirai, Obara, et al., 2021). In our recent study, muscle hypertrophy induced by mechanical overload in mice was significantly reduced when combined with 60 minutes of endurance‐type swimming exercise (Shirai et al., 2025). Resistance training results in skeletal muscle hypertrophy by increasing protein synthesis, and it is regulated by mechanistic target of rapamycin (mTOR) signaling (Miyazaki et al., 2011; Ogasawara et al., 2016; Phillips et al., 1997). Endurance training improves oxidative capacity through mitochondrial adaptations, such as quantity and quality. This adaptation is regulated by the AMP‐activated protein kinase (AMPK)–peroxisome proliferator‐activated receptor gamma coactivator 1‐alpha (PGC1α) pathway (Atherton et al., 2005). AMPK suppresses mTOR activity by phosphorylating tuberous sclerosis complex 2 (TSC2) (Bolster et al., 2002; Inoki et al., 2002). A study in rats found that pharmacologically activated AMPK suppresses mTOR downstream signaling following electrical stimulation (Thomson et al., 2008). This signaling interaction may be the mechanism for the interference effect (Coffey & Hawley, 2017; Shirai et al., 2025). In addition, muscle mass is regulated by net protein balance between protein synthesis and degradation (Phillips et al., 1997). The interference effect may result from increased degradation (Shirai, Hanakita, et al., 2021; Shirai, Obara, et al., 2021). Protein degradation is regulated by the ubiquitin–proteasome system and the autophagy–lysosome system. These systems promote proper protein turnover and maintain muscle quality.

To enhance athletic performance, it is necessary to develop strategies to minimize the interference effect, and HIIT is one such option. However, the molecular basis for the combined effects of different modalities, particularly resistance and high‐intensity interval exercise, remains unclear. Several studies have examined the order of concurrent training and found that molecular adaptation differs depending on the order and combination of modalities (Ogasawara et al., 2014; Shirai et al., 2023), and training adaptations may not be fully obtained depending on the sequence in which they are performed. Many studies have examined CT in combination with resistance and steady‐state endurance exercise, whereas few studies have evaluated physiological adaptations when combined with high‐intensity exercise. Exercise intensity is an important factor in metabolic adaptation, and high‐intensity exercise (e.g., sprint) as well as low‐ to moderate‐intensity exercise is often selected. A meta‐analysis revealed that CT with high‐intensity interval training (HIIT) can minimize interference effects (Vechin et al., 2021). Lee et al. (2024) reported that HIIT does not decrease 10‐week resistance training‐induced hypertrophy and myofibrillar protein synthesis, regardless of the order in which it is performed (Lee et al., 2024). HIIT is time‐efficient because of its specific characteristics of short duration and high intensity, and it has the potential to optimize muscle hypertrophy and metabolic adaptation. However, the molecular basis for the combined effects of different modalities, especially resistance and high‐intensity interval exercise, remains unclear. HIIT, in which high‐intensity exercise is repeated with short rests, improves glycolytic and oxidative metabolic performance simultaneously (Tabata et al., 1997). HIIE induces oxidative adaptation via the AMPK–PGC1α pathway (Bartlett et al., 2012; Gibala et al., 2009). High‐intensity interval exercise (HIIE) also increases mitogen‐activated protein kinase p38 (p38) and Ca2+/calmodulin‐dependent protein kinase II (CaMKII), which are important for mitochondrial biogenesis (Akimoto et al., 2005; Morales‐Alamo et al., 2013; Wu et al., 2002). In addition, HIIE induces hypoxia inducible factor‐1 alpha (HIF‐1α) expression, a regulator of glycolysis, and increases glucose uptake and glycolytic enzyme expression (Abe et al., 2015; Pescador et al., 2010; Semenza, 2010). In CT combined with resistance exercise and HIIE, Apró et al. (2015) found that resistance exercise‐induced phosphorylation of ribosomal protein S6 kinase (S6K1) is not affected by prior HIIE‐induced AMPK activation (Apró et al., 2015). This suggests that a single round of HIIE has little effect on resistance exercise‐induced acute response; however, the effects of these signals on chronic muscle hypertrophy are unclear. It should be noted that AMPK activation returns to basal levels soon after the end of exercise; thus, acute effects must also be considered separately from chronic muscle hypertrophy.

In this study, we examined the effects and mechanisms of the combination of mechanical overload and HIIT by swimming exercise on skeletal muscle hypertrophy by focusing on muscle size and molecular signaling in mice. We have previously confirmed that the interference effect can be assessed by this muscle hypertrophy model and swimming exercise (Shirai et al., 2025). This model allows us to examine the effects of metabolic responses to exercise (especially high intensity) on skeletal muscle hypertrophy while avoiding the effects of excessive ground loading. First, we determined the effects of HIIT on the chronic muscle hypertrophy response. Next, we performed acute experiments, focusing on AMPK expression and mTOR signaling, which are particularly affected by single‐bout HIIE. The results revealed the molecular basis for the combined effects of HIIT on hypertrophied muscle.

## 2 METHODS

### 2.1 Experimental animals

Eight‐week‐old C57BL/6J male mice (Tokyo Laboratory Animals Science Co., Japan) were kept at a room temperature of 22°C ± 2°C, humidity of 55% ± 10%, and a 12‐h light/dark cycle. In this experiment, solid feed for laboratory animals (Oriental Yeast Co., Ltd., MF, Tokyo, Japan) was used. The diet contains approximately 23.1% protein, 5.1% fat, and 71.8% carbohydrate. The feed was provided ad libitum throughout the experimental period, and all animals had free access to water. After the end of the experimental period, the mice were euthanized by cervical dislocation. All experiments were conducted under the approval of the University of Tsukuba Animal Experiment Committee (approval numbers 23‐380 and 24‐274).

### 2.2 Myotenectomy surgery

Mice were divided into three groups (*n* = 6–8): Sham surgery (Sham), myotenectomy‐induced OL (OL), and OL with HIIT by forced swimming (OL + HIIT) in chronic and acute experiments. The OL and OL + HIIT groups underwent myotenectomy surgery as previously described (Fujimaki et al., 2022; Shirai et al., 2025). Under anesthesia with 2.0% isoflurane air inhalation, the synergist tendon of the soleus and gastrocnemius was resected, and plantaris muscle hypertrophy was induced by mechanical overload (OL). For the chronic experiment, the mice were anesthetized, and the plantaris muscle was excised 4 weeks after surgery, weighed, frozen in liquid nitrogen, and stored at −80°C. For the acute experiment, sampling was performed 3 h after surgery.

### 2.3 High‐intensity interval exercise

The HIIE protocol was based on that of previous studies (Abe et al., 2015; Shirai et al., 2023), in which forced swimming exercises were performed in a 60 cm‐deep container filled with water. In this study, the terminology distinguishes between a single exercise bout and the overall training period. A single session of exercise is referred to as high‐intensity interval exercise (HIIE), whereas the chronic, multi‐week intervention consisting of repeated HIIE sessions is termed high‐intensity interval training (HIIT). The water temperature was maintained at 36°C ± 2°C. A weight that was 8% of the body weight was attached to the tail of each mouse, and the mice performed a total of 10 sets of 20 s of swimming and 10 s resting. The load was reset each week after measuring body weight. The exercises were performed five times a week, and sampling was conducted 24 h after the last session was completed in the chronic experiment. In the acute experiment, only the OL + HIIE group was administered HIIE immediately before sampling, whereas all groups had their plantaris muscle harvested and preserved as in the chronic experiment.

### 2.4 Measurement of blood lactate concentration

Blood lactate concentrations were measured to assess the intensity of a single round of exercise. After 1 week of acclimatization, myotenectomy, or sham surgery was performed, and HIIE was conducted only in the OL + HIIE group 3 h after surgery. Before and after exercise, 1 mm of the tail tip of the mice was excised, and blood samples were collected. Lactate Pro 2 (K1000‐01; Arkray) was used for the measurements.

### 2.5 Histological analysis

Plantaris muscles were embedded in Cryomold No. 2 (Tissue‐Tek) filled with OCT compound (Sakura Finetek), frozen in isopentane cooled with liquid nitrogen, and stored at −80°C. Sections were prepared using a cryostat (HM560MV, MICRO EDGE) while cooling to −20°C. The sections were placed onto glass slides (MATSUNAMI) and stored at −20°C. Muscle sections were soaked in Mayer's hematoxylin solution (FUJIFILM Wako Pure Chemical Co.) for 10 min. After washing with warm water, the sections were stained in eosin solution (FUJIFILM Wako Pure Chemical Co.) for 30s. After soaking, the sections were dehydrated in 100% ethanol and mounted using Fluorescent Mounting Media (5570‐0005; SeraCare Life Science Inc). Images of the H&E‐stained muscle sections were captured using an Olympus DP‐74 microscope (Tokyo, Japan) at 20× magnification to determine the transverse area of the myofibers. Cross‐sectional area (CSA) analysis was performed using ImageJ ver. 1.54 (NIH, Bethesda).

### 2.6 Western blot analysis

Western blot analysis was used to quantitate protein expression. The plantaris muscle extract was prepared using RIPA buffer (Nacalai Tesque, Inc.) containing a protease inhibitor cocktail (including EDTA for phosphatase inhibitor) (Nacalai Tesque Inc.) and crushed with a bead crusher (μT‐01, Taitek Corporation). The samples were centrifuged at 15,000 rpm for 20 min at 4°C, and the supernatant was collected. Protein concentration was measured using the Protein Assay Bicinchoninate Kit (06385‐00; Nacalai Tesque Inc.). The samples (10 μg protein) were electrophoresed on a 10% polyacrylamide gel at 150 V for 60 min using a Power Pac‐Universal (Bio‐Rad) power supply. The proteins were transferred to a PVDF membrane (Immun‐Blot PVDF membrane, Bio‐Rad) by a wet method using a tank‐type blotting device (Bio‐Rad) at 30 V for 12 h. Ponceau S (BCL‐PSS‐01; Beacle, Inc.) staining was used as an internal control to ensure that the protein levels were similar. The membrane was blocked with 5% skim milk (FUJIFILM Wako Pure Chemical Co.) in 5% Blocking One in TBST (Nakaraitesque) at room temperature for 1 h and incubated with primary antibody overnight at 4°C. The membrane was washed with TBST and incubated with secondary antibody for 1 h at room temperature. The secondary antibodies included anti‐rabbit IgG, HRP‐linked antibody (1:5000–10,000; 7074S; Cell Signaling Technology) or anti‐mouse IgG, HRP‐linked antibody (1:5000–10,000; 7076S; Cell Signaling Technology). The membrane was washed with TBST, and Immunostar Zeta (FUJIFILM Wako Pure Chemical Co.) or Immunostar LD (FUJIFILM Wako Pure Chemical Co.) was used as the luminescent substrate for detection. The signals were quantified with C‐Digit (LI‐COR Biosciences, Lincoln, NE) and expressed in arbitrary units.

### 2.7 Primary antibodies for western blotting

The following primary antibodies were used for western blot analysis: anti‐protein kinase B (1:3000, #9272, Cell Signaling Technology), anti‐phos‐Akt (1:1000, #9271S; Cell Signaling Technology, Danvers,), anti‐S6K1 (1:1000, #9202; Cell Signaling Technology, Danvers), anti‐phos‐S6K1 (1:1000, sc‐377529, Santa Cruz biochemistry), anti‐S6 ribosomal protein (rpS6) (1:1000, #2217; Cell Signaling Technology), anti‐p‐rpS6 (1:1000, #4858S; Cell Signaling Technology), anti‐4E‐binding protein 1 (4E‐BP1) (1:1000, #9452; Cell Signaling Technology, Danvers), anti‐p‐4E‐BP1 (1:1000, #9459; Cell Signaling Technology, Danvers), anti‐glycogen synthase kinase 3β (GSK3β) (1:1000, #9315; Cell Signaling Technology), anti‐p‐GSK3β (1:1000, #9336; Cell Signaling Technology, Danvers), anti‐extracellular signal‐regulated kinase 1/2 (ERK1/2) (1:1000, #9102; Cell Signaling Technology, Danvers), anti‐phos‐ERK1/2 (1:1000, #9101; Cell Signaling Technology, Danvers), anti‐AMP‐activated kinase (AMPK) (1:500, #2532; Cell Signaling Technology, Danvers), anti‐phos‐AMPK (1:1000, #2531, Cell Signaling Technology, Danvers), anti‐CaMKII (1:1000, #4436: Cell Signaling Technology), anti‐phos‐CaMKII (1:1000, #12716, Cell Signaling Technology), anti‐p38 (1:1000, #9212S, Cell Signaling Technology), anti‐phos‐p38 (1:1000, #9215S, Cell Signaling Technology), anti‐PGC‐1α (1:500; 516,557 Millipore), anti‐HIF‐1α (1:1000, ab1, Abcam), anti‐HIF‐2α (1:1000, NB100–122, Novus Biologicals), anti‐MCT1 (1:1000, sc‐365501, Santa Cruz biochemistry), anti‐FoxO1 (1:1000, #2880, Cell Signaling Technology), anti‐phos‐FoXO1 (1:1000, #2599, Cell Signaling Technology), anti‐Ubiquitin (1:1000, #3936, Cell Signaling Technology), anti‐MuRF1 (1:1000, ab172479, Abcam), anti‐MAFbx (1:1000, ab168372, Abcam), anti‐p62 (1:1000, PM045, MBL), and anti‐LC3 (1:1000, #4108S, Cell Signaling Technology).

### 2.8 Statistical analysis

Data were analyzed using GraphPad 10.0 software (La Jolla, CA, USA). The values are reported as means ± standard deviation (SD) and individual values. All data were analyzed by a one‐way (group) ANOVA. When a significant *p* value was obtained, statistical significance (post hoc test) was calculated using Tukey's method. Statistical significance was set at *p* < 0.05.

## 3 RESULTS

### 3.1 Effect of chronic high‐intensity interval exercise on the plantaris muscle hypertrophic response

#### 3.1.1 Body weight and muscle size

**FIGURE 1:**
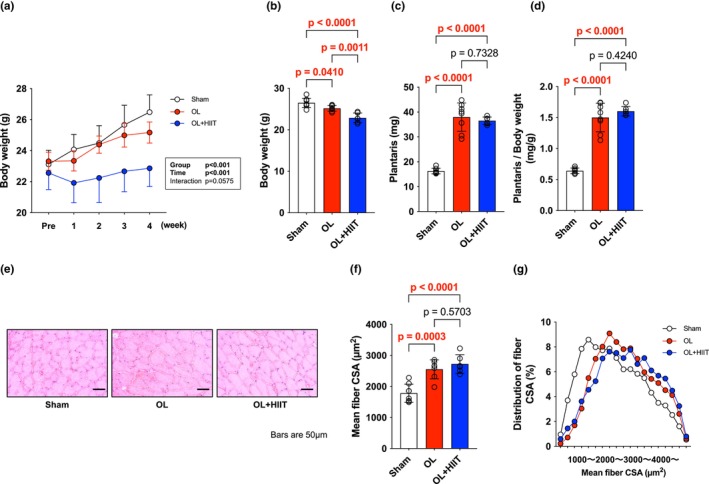
High‐intensity interval exercise does not affect overloaded muscle size. (a) Body weight during 4 weeks of intervention. (b) Body weight at the end of the intervention. (c) Plantaris muscle wet weight. (d) Plantaris muscle wet weight per body weight. (e) H&E‐stained images (f) Mean fiber CSA. (g) Distribution of fiber CSA. All data are expressed as means ± SD and individual values (*n* = 6–8). Significant differences were assessed by a one‐way ANOVA followed by Tukey's multiple comparison test. Bar showing significant differences between the corresponding groups.

After a 4‐week intervention, the OL group showed significantly lower weight gain compared with that in the Sham group (*p* = 0.0410), and the OL + HIIT group showed significantly lower weight compared with that in the Sham and OL groups (*p* < 0.0001, *p* = 0.0011) (Sham: 26.49 ± 1.13 g; OL: 25.17 ± 0.68 g; OL + HIIT: 22.87 ± 1.17 g). Plantaris muscle wet weight was significantly higher in the OL and OL + HIIT groups compared with that in the Sham group (*p* < 0.0001), with no significant differences between the OL and OL + HIIT groups (*p* = 0.7328) (Figure 1c). Relative plantaris muscle weight was also significantly higher in the OL and OL + HIIT groups compared with that in the Sham group (*p* < 0.0001). Mean fiber CSA of the plantaris muscle was significantly increased in the OL (*p* = 0.0003) and OL + HIIT groups (*p* < 0.0001) compared with that in the Sham group. No significant difference was observed between the OL and OL + HIIT groups (*p* = 0.5703) (Figure 1e). The distribution of CSA revealed a shift to the right in the OL and OL + HIIT groups (Figure 1f).

#### 3.1.2 mTOR signaling‐related proteins

**FIGURE 2:**
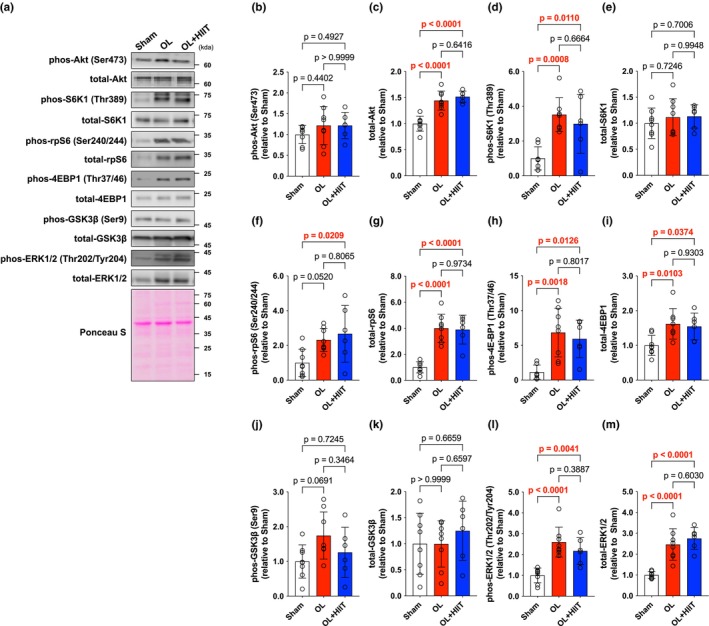
High‐intensity interval training does not affect mTOR signaling‐related proteins. (a) Band images. (b) phos Akt (Ser473). (c) total Akt. (d) phos S6K1 (Thr389). (e) total S6K1. (f) phos rpS6 (Ser240/244). (g) total rpS6. (h) phos 4E‐BP1 (Thr37/46). (i) total 4E‐BP1. (j) phos GSK3β (Ser9). (k) total GSK3β. (l) phos ERK (Thr202/Tyr204). (m) total ERK1/2. All data are expressed as means ± SD and individual values (*n* = 6–8). Significant differences were assessed by a one‐way ANOVA followed by Tukey's multiple comparison test. Bar showing significant differences between the corresponding groups.

Akt phosphorylation was unchanged, whereas total Akt levels were significantly increased in the OL (*p* < 0.0001) and OL + HIIT (*p* < 0.0001) groups (Figure 2b,c). S6K1 phosphorylation was significantly increased in the OL (*p* = 0.0008) and OL + HIIT (*p* = 0.0110) groups compared with that in the Sham group (Figure 2d). Total levels of S6K1 were unchanged (Figure 2e). rpS6 phosphorylation was increased in the OL + HIIT (*p* = 0.0209) group compared with that in the Sham group (Figure 2f). Total levels of rpS6 were increased in the OL (*p* < 0.0001) and OL + HIIT (*p* < 0.0001) groups compared with that in the Sham group (Figure 2g). 4E‐BP1 phosphorylation was significantly increased in the OL (*p* = 0.0018) and OL + HIIT groups (*p* = 0.0126) compared with that in the Sham group (Figure 2h). Total levels of 4E‐BP1 were significantly increased in the OL (*p* < 0.0103) and OL + HIIT (*p* = 0.0374) groups compared with that in the Sham group (Figure 2i). GSK3β were unchanged in both phosphorylation and total amount (Figure 2j,k). ERK1/2 phosphorylation was increased in the OL (*p* < 0.0001) and OL + HIIT (*p* = 0.0041) groups compared with that in the Sham group (Figure 2l). Total levels of ERK1/2 were also significantly increased in the OL (*p* < 0.0001) and OL + HIIT (*p* < 0.0001) groups compared with that in the Sham group (Figure 2m). Phosphorylation and total levels No significant differences were observed between the OL and OL + HIIT groups for any of the proteins examined.

#### 3.1.3 Oxidative/glycolytic metabolism‐related proteins

**FIGURE 3:**
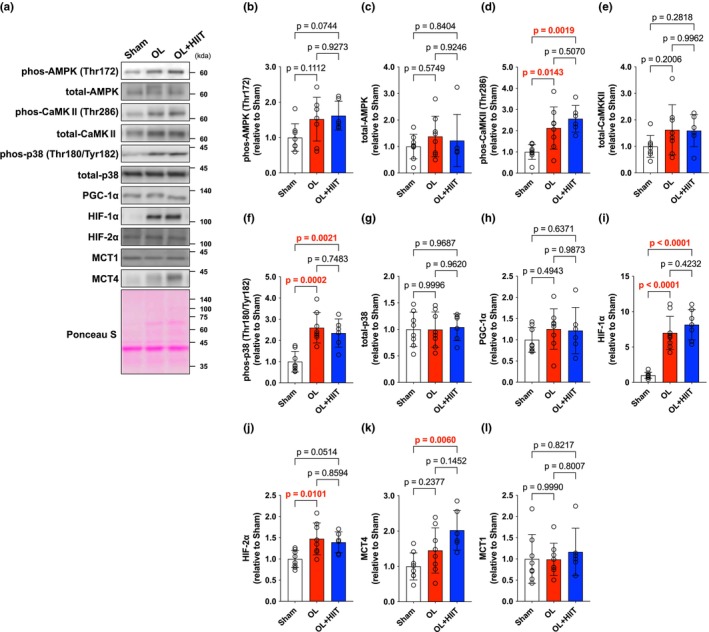
Mechanical overload and high‐intensity interval training increase oxidative/glycolysis‐related proteins. (a) Band images. (b) phos AMPK (Thr172). (c) total AMPK. (d) phos CaMKII (Thr286). (e) total CaMKII. (f) phos p38 (Thr180/Tyr182). (g) total p38. (h) PGC‐1α. (i) HIF‐1α. (j) HIF‐2α. (k) MCT1. (l) MCT4. All data are expressed as means ± SD and individual values (*n* = 6–8). Significant differences were assessed by a one‐way ANOVA followed by Tukey's multiple comparison test. Bar showing significant differences between the corresponding groups.

No difference was observed in AMPK phosphorylation and total levels (Figure 3b,c). CaMKII phosphorylation was significantly higher in the OL (*p* = 0.0143) and OL + HIIT (*p* = 0.0019) groups compared with that in the Sham group (Figure 3d). Whereas total levels of CaMKII were unchanged (Figure 3e). p38 phosphorylation was increased in the OL (*p* = 0.0021) and OL + HIIT groups (*p* = 0.0002) compared with that in the Sham group (Figure 3f). Total levels of p38 were unchanged (Figure 3g). No difference was observed in PGC‐1α expression (Figure 3h). HIF‐1α expression was significantly higher in the OL (*p* < 0.0001) and OL + HIIT (*p* < 0.0001) groups compared with that in the Sham group (Figure 3i). HIF‐2α and MCT1 expression were unchanged after the 4‐week intervention (Figure 3j,k), whereas MCT4 expression was significantly increased in the OL + HIIT group compared with that in the Sham group (*p* = 0.0060) (Figure 3l).

#### 3.1.4 Protein degradation‐related proteins

**FIGURE 4:**
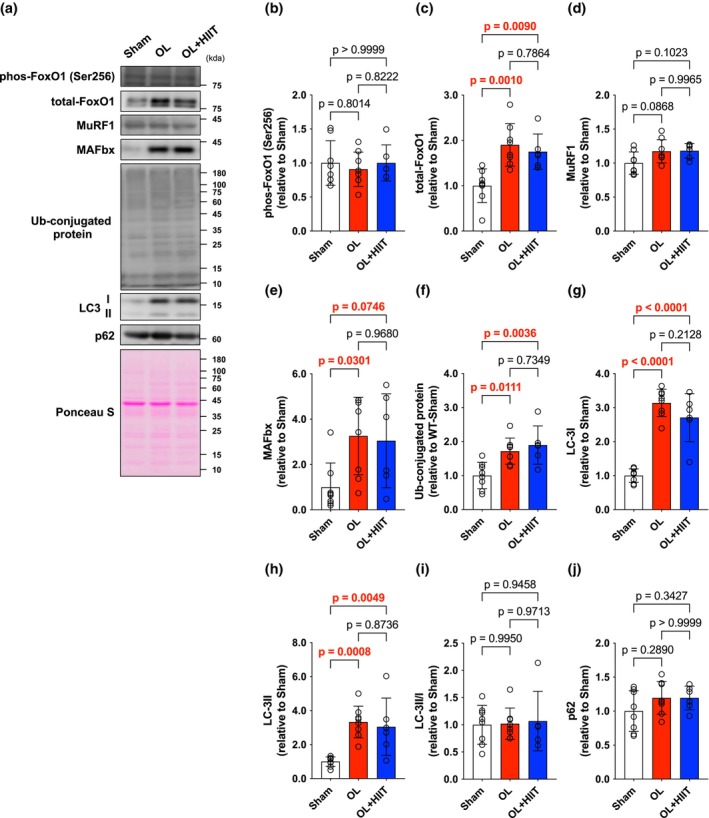
High‐intensity interval training does not enhance protein degradation induced by mechanical overload. (a) Band images. (b) phos FoXO1 (Ser256). (c) Total FoXO1. (d) MuRF1. (e) MAFbx. (f) Ubiquitin‐conjugated protein. (g) LC‐3I. (h) LC‐3II. (i) LC‐3II/I ratio. (j) p62. All data are expressed as means ± SD and individual values (*n* = 6–8). Bar showing significant differences between the corresponding groups.

After a 4‐week intervention, there were no significant changes in FoxO1 phosphorylation (Figure 4b). However, total levels of FoxO1 were increased in OL (*p* = 0.0010) and OL + HIIT groups (*p* = 0.0090) compared with those in the Sham group (Figure 4c). MuRF1 expression was unchanged among the groups (Figure 4d). MAFbx protein expression was significantly higher in the OL (*p* = 0.0301) groups compared with those in the Sham group; however, no difference was observed in the OL and OL + HIIT groups (*p* = 0.9680) (Figure 4e). Ubiquitin‐conjugated protein was similarly increased in the OL (*p* = 0.0111) and OL + HIIT (*p* = 0.0036) groups compared with those in the Sham group (Figure 4f). LC3 I and II expressions were significantly increased in the OL (LC3I: *p* < 0.0001; LC3II: *p* = 0.0008) and OL + HIIT (LC3I: *p* < 0.0001; LC3II: *p* = 0.0049) groups, but the LC3II/I ratio was similar in all the groups (Figure 4g–i). The expression of p62 was not affected by OL or HIIT (Figure 4j).

### 3.2 Effect of acute high‐intensity interval exercise on protein synthesis‐related signals induced by mechanical overload

**FIGURE 5:**
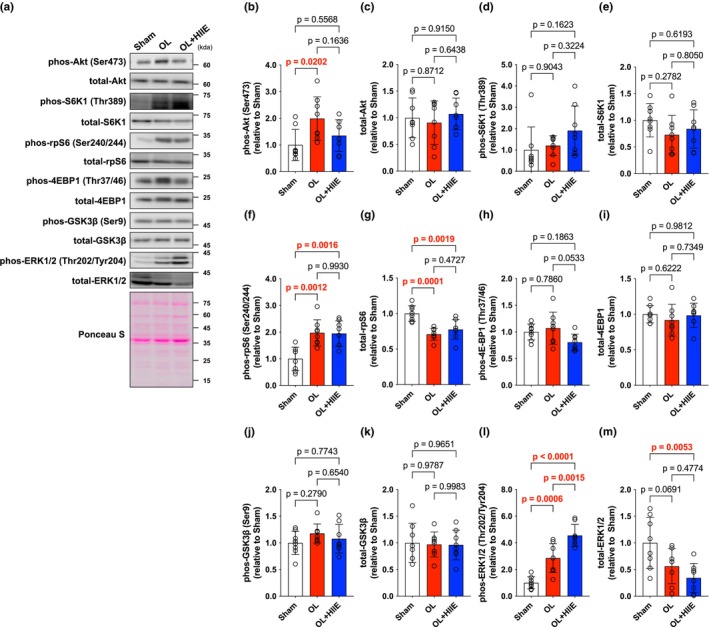
High‐intensity interval exercise does not interfere with mTOR signaling‐related proteins. (a) Band images. (b) phos Akt (Ser473). (c) total Akt. (d) phos S6K1 (Thr389). (e) total S6K1. (f) phos rpS6 (Ser240/244). (g) total rpS6. (h) phospho 4E‐BP1 (Thr37/46). (i) total 4E‐BP1. (j) phos GSK3β (Ser9). (k) total GSK3β. (l) phos ERK1/2 (Thr202/Tyr204). (m) total ERK1/2. All data are expressed as means ± SD and individual values (*n* = 8). Significant differences were assessed by a one‐way ANOVA followed by Tukey's multiple comparison test. Bar showing significant differences between the corresponding groups.

Blood lactate concentration after acute exercise was higher in the OL + HIIE group compared with that in the Sham (*p* = 0.0012) and OL (*p* = 0.0002) groups (Sham: 3.88 ± 1.73–4.2 ± 1.40; OL: 4.4 ± 1.0–3.25 ± 0.43; OL + HIIE: 3.79 ± 1.54–11.66 ± 7.91). Akt phosphorylation was increased in the OL group compared with that in the Sham group (*p* = 0.0202) (Figure 5b). Total Akt levels were unchanged in any groups (Figure 5c). S6K1 phosphorylation was higher in the OL + HIIE (*p* = 0.0469) group compared with that in the Sham group (Figure 5d). rpS6 phosphorylation was higher in the OL (*p* = 0.0012) and OL + HIIE (*p* = 0.0016) groups compared with that in the Sham group, whereas there was no difference in the OL and OL + HIIE groups (*p* = 0.9930) (Figure 5f). Total levels of rpS6 were significantly decreased in OL (*p* = 0.0001) and OL + HIIE (*p* = 0.0019) compared with that in the Sham group (Figure 5g). 4E‐BP1 and GSK3β were unchanged in both phosphorylation and total amount (Figure 5h–k). ERK1/2 phosphorylation was higher in the OL (*p* = 0.0006) and OL + HIIE groups (*p* < 0.0001) with that in the Sham group (Figure 5l). The OL + HIIE group exhibited higher expression compared with the OL group (*p* = 0.0015) (Figure 5l). Total levels of ERK1/2 were decreased in the OL + HIIE group compared with that in the Sham group (*p* = 0.0053) (Figure 5m).

**FIGURE 6:**
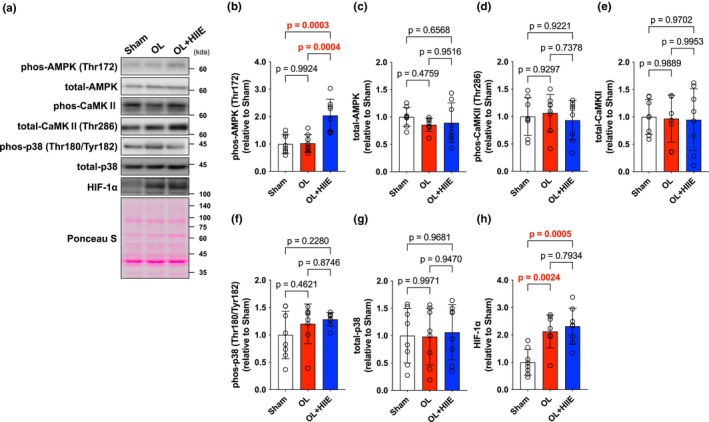
Effects of high‐intensity interval exercise on metabolism‐related proteins. (a) Band images. (b) phos AMPK (Thr172). (c) total AMPK. (d) phos CaMKII (Thr286). (e) total CaMKII. (f) phos p38 (Thr180/Tyr182). (g) total p38. (h)HIF‐1α. All data are expressed as means ± SD and individual values (*n* = 8). Significant differences were assessed by a one‐way ANOVA followed by Tukey's multiple comparison test. Bar showing significant differences between the corresponding groups.

Single‐bout HIIE promoted AMPK phosphorylation in the OL + HIIE group compared with that in the Sham (*p* = 0.0003) and OL (*p* = 0.0004) groups, whereas total levels were unchanged (Figure 6a,b). No changes were observed for CaMKII and p38 in both phosphorylation and total amount (Figure 6d–g). HIF1‐α expression was significantly higher in the OL (*p* = 0.0024) and OL + HIIT (*p* = 0.0005) groups compared with that in the Sham group (Figure 6h).

## 4 DISCUSSION

In this study, we clarified the effect of CT composed of mechanical overload and HIIT on muscle hypertrophy and its molecular basis in the chronic and acute response. After 4‐week combined OL and HIIT intervention, plantaris muscle wet weight and CSA were higher in the OL and OL + HIIT groups compared with that in the Sham group. Notably, the addition of HIIT to OL did not affect plantaris muscle hypertrophy. The phosphorylation of S6K1, rpS6, and 4E‐BP1 downstream of the mTOR pathway was higher in the OL and OL + HIIT groups compared with that in the Sham group, indicating that HIIT does not decrease adaptation associated with OL. These results differ from our previous reports of attenuated muscle hypertrophic adaptation with low‐intensity exercise (Shirai et al., 2025).

Previous studies have indicated that HIIT induces oxidative metabolic adaptation via AMPK activity (Bartlett et al., 2012; Gibala et al., 2009; Pengam et al., 2023; Pirani et al., 2023). AMPK negatively regulates mTOR signaling and may be a factor in the interference effects (Coffey & Hawley, 2017). In the present study, acute experiments showed a significant increase in AMPK phosphorylation in the OL + HIIE groups; however, the increase in rpS6 and ERK phosphorylation by OL was not immediately suppressed following a single bout of HIIE. This suggests that HIIE‐induced AMPK activation has little acute effect on mTOR signaling. (However, the long‐term impact of repeated HIIE sessions on mTOR‐related anabolic pathways remains unclear and warrants further investigation. It is also necessary to consider the potential interaction between AMPK and other signaling molecules involved in muscle adaptation). Although AMPK has been described in previous studies to inhibit mTOR signaling, this previous validation was based on pharmacological activation of AMPK (Bolster et al., 2002; Thomson et al., 2008). Studies using AMPK‐dominant negative or knockdown mice have shown that AMPK has minimal impact on muscle contraction or mechanical overload‐induced mTOR signaling (Kido et al., 2021; Knudsen et al., 2020). Additionally, other reports indicate that AMPK activation by endurance exercise following resistance exercise, or by high‐intensity interval exercise preceding resistance exercise, does not inhibit mTOR signaling (Apró et al., 2013, 2015). These findings suggest that AMPK activation induced by exercise may not play a major role in regulating mTOR signaling or mediating the interference effect, although its precise contribution remains to be fully elucidated. Furthermore, we previously examined the combined effects of OL and treadmill running (Shirai, Hanakita, et al., 2021; Shirai, Obara, et al., 2021). The results indicated that mTOR signaling was not suppressed by running, whereas ubiquitinated proteins and oxidative stress were increased compared with that in the OL‐only group (Shirai, Hanakita, et al., 2021; Shirai, Obara, et al., 2021). This suggests that factors other than AMPK promote its degradation and are responsible for the interference effect.

Muscle mass is determined not only by protein synthesis, but also by degradation (Phillips et al., 1997). In this study, we investigated the expression of proteins associated with the ubiquitin–proteasome and autophagy–lysosome pathways to evaluate muscle protein degradation in response to OL and HIIT. Four‐week intervention increased ubiquitin‐conjugated proteins in the OL and OL + HIIT groups compared with that in the Sham group, whereas no difference was observed between the OL and OL + HIIT groups. This suggests that degradation was enhanced in response to a drastic hypertrophic response and increased energy demand induced by OL but was not affected by HIIT. MAFbx expression was higher in the OL and OL + HIIT groups compared with that in the Sham group. No difference was observed in MuRF1 expression. We acknowledge that a comprehensive analysis of proteolytic flux, including proteasome and deubiquitinating enzyme activity, was not performed in this study. Our key finding, however, is that the addition of HIIT did not cause further changes in markers for the ubiquitin–proteasome system (ubiquitinated proteins, MAFbx) or the autophagy‐lysosome system (p62) compared to the OL group alone. This suggests that HIIT did not enhance protein degradation pathways beyond the effect of mechanical overload itself. We also examined the autophagy–lysosome system. The expression of p62, which represents a target for autophagic degradation, did not change between the groups, whereas LC3‐I and II were increased in the OL and OL + HIIT groups compared with that in the Sham group. This indicates that autophagy proceeded appropriately, and intracellular homeostasis was maintained. Skeletal muscle integrity is maintained by the protein degradation system (Osana et al., 2025). Therefore, even if some degraded substrates increase, this is associated with drastic hypertrophy and increased energy demand, which is considered appropriate turnover. HIIT did not significantly affect these mechanisms and did not appear to be involved in the negative regulation of muscle mass. In summary, proteolysis does not appear to be influenced by HIIT. However, this may not apply to other modalities of exercise, and further investigation is required to clarify whether protein degradation pathways contribute substantially to the interference effect.

We quantified the MAPK pathway, which is involved in numerous adaptations in response to mechanical or metabolic stress. p38 is upregulated by HIIE along with increased expression of PGC1α (Bartlett et al., 2012; Gibala et al., 2009). The MAPK pathway is activated by resistance exercise or mechanical overload in rodents (Huey, 2006; Williamson et al., 2003). In particular, ERK1/2 is involved in protein synthesis through phosphorylation of TSC2 and activates Rheb, which is a key regulator of mTORC1 (Miyazaki et al., 2011; Miyazaki & Takemasa, 2017). In the chronic experiment, ERK1/2 (p42/44 MAPK) and p38 phosphorylation were higher in the OL and OL + HIIT groups after a 4‐week intervention, without enhancement or interference by HIIT. In acute experiments, upregulation of ERK1/2 induced by OL was further enhanced by a single bout of HIIE. Although HIIE activated AMPK immediately after exercise, it did not reduce the expression of proteins downstream of mTORC1. This response may reflect an additive effect of HIIE on OL through ERK signaling, a known positive regulator of mTORC1, suggesting that HIIE‐induced ERK activation might attenuate the interference effect.

Intracellular Ca^2+^ may also regulate muscle hypertrophy by upregulating the LMCD1 or PGC‐1α4 pathway (Ferreira et al., 2019; Mammucari et al., 2015). We demonstrated that the increase in phosphorylation of CaMKII reflects Ca^2+^ accumulation in the OL and OL + HIIT groups, but it is not enhanced by HIIT. Therefore, metabolic stress related to HIIT does not affect protein synthesis.

The difference between the present results and those of our previous study using long‐duration, moderate‐intensity exercise may reflect differences in metabolic responses. The HIIE protocol applied in the current study primarily relies on glycolysis for ATP production. Previous work has shown that inhibition of glycolysis suppresses electrically evoked protein synthesis (Suginohara et al., 2021). This suggests that metabolites produced during glycolysis may play a crucial role in regulating protein synthesis. Lactate, a major byproduct of glycolysis, is one such candidate metabolite that has recently been proposed to act as a signaling molecule. Lactate has been shown to increase myotube diameter in C2C12 cells (Ohno et al., 2018; Oishi et al., 2015) and to stimulate mTOR signaling in mice (Cerda‐Kohler et al., 2018), supporting a potential role in muscle hypertrophy and protein synthesis. However, exogenous administration of lactate does not promote muscle growth in either mice or humans (Liegnell et al., 2020; Shirai et al., 2022), and its role as a signaling factor remains unclear. Notably, blood flow restriction, which increases endogenous lactate levels, has been reported to enhance S6K1 phosphorylation and protein synthesis (Fujita et al., 2007), suggesting that intracellular lactate may contribute to these effects. In the present study, both blood lactate levels following a single bout of HIIE and MCT4 protein expression were significantly elevated in the OL + HIIT group, suggesting increased lactate accumulation within muscle. These findings raise the possibility that glycolytic activation during HIIE may mitigate the interference effect, either via lactate or other glycolysis‐related metabolites. Further work involving pharmacological inhibition of glycolysis or genetic targeting of relevant metabolic pathways is needed to clarify the mechanisms involved. These are only a small fraction of candidates, and in fact, there is multi‐organ crosstalk during exercise. It is possible that these metabolites known as “Exerkines” play a major role in the adaptation of exercise combinations (Sato et al., 2022; Thyfault & Bergouignan, 2020). A comprehensive analysis focusing on metabolites will clarify their role.

This study had some limitations. In the present study, we focused on the hypertrophic response when combined with HIIT. Based on the study design, which emulates CT, the volume of exercise is different because of the added workload in the OL + HIIT group. Such high‐intensity exercise may also result in a different energy expenditure/intake balance and resting behavior (Funabashi et al., 2024). Future studies should focus on their involvement in skeletal muscle adaptation. We could not evaluate the protein synthesis rate using methods such as SUnSET (Goodman et al., 2011). This will be needed to further understand the hypertrophic response directly. We only evaluated the regulation of muscle mass; however, the combined effect of OL and HIIT on contractile and endurance capacity was not revealed. In addition, mitochondrial function (e.g., respiratory) should be examined to assess HIIT‐dependent adaptation. Future studies are needed to assess the HIIT‐only group. Finally, we note that the generalizability of our findings is limited by the specific muscle and sex we used in this study. The present study utilized the plantaris muscle, which predominantly consists of fast‐twitch fibers. However, differences in adaptation may occur depending on muscle fiber type. Furthermore, in line with many previous studies, including our own concurrent training experiments, we exclusively used male mice. As sex differences in muscle hypertrophy and metabolic responses related to interference effects can arise due to hormonal and genetic factors, further investigation is needed in this regard.

## 5 CONCLUSION

The combination of OL and HIIT does not interfere with skeletal muscle hypertrophy compared with OL alone. mTOR signaling is not suppressed by HIIE, either in the acute or chronic state. This may be attributed to the short duration and high intensity of the exercise, which suppresses the interference effect. The acute experiment raises the question of AMPK's involvement in the interference effect; however, further studies are needed regarding the mechanism of the interference effect.

## AUTHOR CONTRIBUTIONS

H.S., T.S., and K.U. conceived and designed research; H.S., T.S., K.U., R.I., T.I., R.T., and S.S. performed experiments; H.S., and T.S. analyzed data; H.S. and T.S. prepared the figure; H.S. wrote the manuscript; H.S., T.S., K.U., T.I, and T.T. revised the manuscript; all authors approved the final version of the manuscript.

## FUNDING INFORMATION

This work was supported by the Japan Society for the Promotion of Science KAKENHI (Grant Nos: 20J15222, 22K17692 and 23KJ2060 to T.S; 23H03266 to T.T) and the NAKATOMI foundation to T.S.

## CONFLICT OF INTEREST STATEMENT

The authors declare no conflicts of interest.

## ETHICS STATEMENT

All experiments were conducted with the approval of the University of Tsukuba Animal Experiment Committee (approval numbers 23‐380, 24‐274) and in accordance with national guidelines.

## Data Availability

Data will be made available upon reasonable request.
